# Medical Convention of Ohio

**Published:** 1843-04

**Authors:** 


### MEDICAL CONVENTION OF OHIO.

We would remind our readers that, according to adjournment, this body meets in Lancaster, Ohio, on the second Monday in May—the 8th day of the month. We anticipate an unusually interesting meeting, as at the last session, held in Cincinnati, and which we had the pleasure of attending, many subjects of interest were referred to the coming one.

We acknowledge the reception of the "Proceedings" of the fifth (last) session of the convention, which multiplied engagements have hitherto prevented us from noticing. We observe that it contains two papers, one "On the Topagraphy and Diseases of Scioto County, Ohio," by Dr. G. S. B. Hempstead of Portsmouth; the other "On the Causes and Treatment of Milk-sickness," by Dr. John Dawson, of Greene County. The rule adopted by the "Censors" (in the absence of any definite instructions from the convention) cut off all the other papers placed in their hands. This rule seems to us in the main correct, as also do the suggestions made by this committee in reference to the proper objects of attention on the part of members of the convention. A few such papers as that of Dr. Hempstead, would give the body a character abroad, which volumes of wire-drawn speculations on old and trite subjects would never create for it. We shall refer to this paper hereafter. C.

